# Analysis of inequality in maternal and child health outcomes and mortality from 2000 to 2013 in China

**DOI:** 10.1186/s12939-017-0558-2

**Published:** 2017-04-20

**Authors:** Yanting Li, Yimin Zhang, Shuai Fang, Shanshan Liu, Xinyu Liu, Ming Li, Hong Liang, Hua Fu

**Affiliations:** 1 School of Public Health, and the Key Laboratory of Public Health Safety, Ministry of Education, Fudan University, Shanghai, 200032 China; 2 Pudong Institute for Health Development, Shanghai, 200129 China; 3 School of Social Development and Public Policy, Fudan University, Shanghai, 200433 China; 4 Health and Family Planning Commission of Pudong new district, Shanghai, 200125 China

**Keywords:** Maternal and child health, Health outcome inequality, Death causes constituent

## Abstract

**Background:**

Inequality in maternal and child health seriously hinders the overall improvement of health, which is a concern in both the United Nations Sustainable Development Goals (SDGs) and Healthy China 2030. However, research on the equality of maternal and child health is scarce. This study longitudinally assessed the equality trends in China’s maternal and child health outcomes from 2000 to 2013 based on place of residence and gender to improve the fairness of domestic maternal and child health.

**Methods:**

Data on China’s maternal and child health monitoring reports were collected from 2000 to 2013. Horizontal and vertical monitoring were performed on the following maternal and child health outcome indicators: incidence of birth defects (IBD), maternal mortality rate (MMR), under 5 mortality rate (U5MR) and neonatal mortality rate (NMR). The newly developed HD*Calc software by the World Health Organization (WHO) was employed as a tool for the health inequality assessment. The between group variance (BGV) and the Theil index (T) were used to measure disparity between different population groups, and the Slope index was used to analyse the BGV and T trends.

**Results:**

The disparity in the MMR, U5MR and NMR for the different places of residence (urban and rural) improved over time. The BGV (Slope BGV = -32.24) and T (Slope T = -7.87) of MMR declined the fastest. The gender differences in the U5MR (Slope BGV = -0.06, Slope T = -0.21) and the NMR (Slope BGV = -0.01, Slope T = 0.23) were relatively stable, but the IBD disparity still showed an upward trend in both the place of residence and gender strata. A decline in urban-rural differences in the cause of maternal death was found for obstetric bleeding (Slope BGV = -14.61, Slope T = -20.84). Improvements were seen in the urban-rural disparity in premature birth and being underweight (PBU) in children under 5 years of age. Although diarrhoea and pneumonia decreased in the U5MR, no obvious gender-based trend in the causes of death was observed.

**Conclusion:**

We found improvement in the disparity of maternal and child health outcomes in China. However, the improvements still do not meet the requirements proposed by the Healthy China 2030 strategy, particularly regarding the rise in the IBD levels and the decline in equality. This study suggests starting with maternal and child health services and focusing on the disparity in the causes of death in both the place of residence and gender strata. Placing an emphasis on health services may encourage the recovery of the premarital check and measures such as prenatal and postnatal examinations to improve equality.

## Background

Maternal and child health-related indicators comprise two of the eight development goals in the United Nations Millennium Development Goals (MDGs) (i.e., reducing child mortality and improving maternal health) [1]. This plan aims to reduce child mortality and improve maternal health (1990–2015) by calling for the following changes: a reduction by two-thirds in the mortality of children under 5 years of age, a reduction of three-quarters in maternal mortality, and universal reproductive health by 2015. In September 2000, China officially became a signatory to the MDGs and included women and children as the focus groups in the Healthy China 2030 Planning Outline. In 2010, the World Health Organization (WHO) evaluated the regional and worldwide achievements of the MDGs [2]. A scoring system based on 10 indicators was employed, and the results showed that the worldwide achievements in the MDGs were not satisfactory. Specifically, three of the four maternal and child health-related evaluation indicators failed to show adequate progress. As stated in the final report of the United Nations (the *Report of MDGs 2015*), the MDGs have not been fully achieved, and inequality still persists. This statement was followed by the target of the Sustainable Development Goals (SDGs) (i.e., reducing cases such as international inequalities). In September 2015, the member states of the MDGs re-signed the SDGs, including Reducing Health Inequality at Home and Abroad [3].

Equality-related research has developed rapidly over the past 30 years. In the 1980s, only approximately one dozen papers on equity were published each year [4]. By 2015, a total of 3521 Chinese and English documents on equity/equality in health were published, indicating that policy makers, project sponsors and non-governmental organizations paid more attention to the need for increased research in equality in health. Many of the policy statements and findings from the WHO strongly call for a reduction in the disparities in maternal and child health between countries and different socioeconomic groups within countries [5–7]. The World Development Report 2006 published by the World Bank noted that the inequality of opportunity between countries was staggering, not only in terms of inequality in survival opportunities, but also in education, health and the use of infrastructure and other public services [8]. Relevant foreign studies have shown that the coverage rate and service quality of antenatal care are significantly higher in urban areas than in rural areas [9, 10]. Women with higher levels of education are more likely to choose caesarean delivery for childbirth and receive more antenatal care [11]. Additionally, children in poor households are more prone to growth retardation, being underweight, anaemia, and diarrhoea [12]. Health equality in practice has been actively explored in foreign countries, and a series of related measures have been formulated; these measures have mainly included changes in the public health service model by integrating health resources, expanding health service areas and strengthening supervision and management. A series of measures has contributed to the improvement of health equality [13]. One study has shown a distinct difference in women’s and children’s health in Asian countries, with up to 33-fold differences in the under-five mortality rate (U5MR) between Japan and Afghanistan and 67-fold differences in the maternal mortality rate (MMR) [14]. The prevalence of this health inequality was a concern of Marie-Paule Kieny, Deputy Director-General of the WHO [15], who noted that the current primary task was to ensure the continued health improvement of vulnerable groups by monitoring health inequalities in developing countries.

The condition of maternal and child health equality is not optimistic in China, which is the largest developing country. China has fully committed to the MDGs and has achieved 13 MDG targets over the past 15 years. The under-five child mortality rate dropped from 61.0% in 1991 to 12.0% in 2013, the gap in the child mortality rate between urban and rural areas narrowed from 1:3.4 to 1:2.4, the maternal mortality rate declined from 88.8 per 100,000 in 1990 to 23.2 per 100,000 in 2013, and the maternal mortality rate between rural and urban areas narrowed from 1:2.2 to 1:1.1 [16]. However, an obvious gap in key maternal and child health indicators still exists between urban and rural areas and among different regions. A notable difference remains in the mortality rates of newborns and children under 5 years of age between rural and urban areas. Differences can also be seen among various regions. According to data from the China Health Statistical Yearbook, the MMR and U5MR in the western region are 2.6 and 3.1 times higher, respectively, than the rates in the eastern region [17]. A study in China’s poorest rural areas showed infant mortality rates (IMRs) that were approximately 5 times higher in China’s poorest rural areas compared to the richest regions (123 and 26 per thousand in the poorest and richest areas, respectively) [18]. China’s 10-year follow-up survey showed that the U5MR among the poorest people (accounting for one-fifth of the population) was 6 times the U5MR among the richest people (accounting for one-fifth of the population). The U5MR was only 10% in the richest big cities but was 64% in poor rural areas. An approximately three-fold difference was found in the malnutrition rate of children under 5 years of age between urban and rural areas. The incidence of childhood stunting was 17.3% in rural areas and only 4.9% in urban areas, and the incidence of low birth weight infants was 9.3% in rural areas and only 3.1% in urban areas [19].

Despite inequalities in international and domestic maternal and child health, research in this area is still relatively scarce. After literature searches of the Web of Science, Engineering Village, Wiley Online Library, Science Direct, Springer Link, VIP, Wangfang Data, and CNKI databases, we found only 30 articles focusing on the equality of maternal and child health. Moreover, studies related to the theory of equality of maternal and child health services started later in China. An analysis of the Chinese literature indicated that related studies gave priority to a cross-sectional design (e.g., a study of the current situation and the equity of the intervening measures to promote maternal and child health coverage in China [20], an analysis of the MMR, child mortality and impact factor levels [21], and an analysis of the equality of basic public health services between different regions [22]). Fewer longitudinal studies have been performed (e.g., an analysis of the equality allocation of maternal health care resources in China between 2005 and 2009 [23]); moreover, these studies have focused on analysing the health input dimensions and the disparity in the MMR in urban and rural areas between 1996 and 2006 [24]. Moreover, studies on the developing equality trend for only one outcome indicator lack a comprehensive analysis of the equality trend in multiple indicators of maternal and child health outcomes and the causes of death.

In this study, several representative indicators of maternal and child health outcomes were selected based on place of residence and gender strata; from these data, a longitudinal assessment was conducted on equality and the tendencies of China’s maternal and child health outcomes with a distributive difference analysis on the maternal death causes and causes of death in children under 5 years of age. The aim of the study is to suggest strategies and measures to improve the equality of maternal and child health outcomes in China.

## Methods

### Data collection

The data were collected from China’s 2000–2013 maternal and child health surveillance system, which was nationally and originally collected from maternal and child death records. The data were reported from maternity and child care institutions in 336 locations and were investigated by level. We selected the following maternal and child health outcome indicators: incidence of birth defects (IBD), MMR, under 5 mortality rate (U5MR), and neonatal mortality rate (NMR). We calculated the estimated values, sample size and standard error for the causes of death based on location (urban and rural areas) and gender.

### Interviews

We interviewed several officials in charge of the maternal and child health department of the health bureau in Hubei, Qinghai, China, to explain the data analysis findings.

### Measurement indicators and definitions

The indicators in the questionnaire were selected based on a comprehensive literature review and expert consultation. Ultimately, disease and death outcomes were selected to explain the equality of maternal and child health outcomes in China. The disease outcome dimension included the IBD, which referred to the ratio of the number of annual IBD cases to the number of births that occurred in that year in the jurisdiction. The death outcome dimension included the MMR, NMR, and U5MR; the MMR refers to maternal mortality due to all causes from pregnancy up to 42 days after giving birth (except accidents), the NMR refers to live births born after 28 weeks of pregnancy (i.e., the ratio of deaths within 28 days after birth to the number of births), and the U5MR refers to the probability of death before the age of 5 years in children born in a given year. Focused analyses were performed on the cause of maternal mortality and the cause of under-five mortality. In this study, the causes of maternal death included postpartum haemorrhage (PH), puerperal infection (PI), pregnancy-induced hypertension (PIH), medical complications (MC) and amniotic fluid embolism (AFE); the causes of death in children under the age of 5 years included premature birth and being underweight (PBU), birth asphyxia (BA), diarrhoea, and pneumonia, as well as others. The maternal and child health outcomes included in this study are negative indicators, with index definitions and formulas consistent with the definitions and contents in the monitoring and investigation lists of national maternity and child care institutions.

### Statistical analysis

In the current study, the latest HD*Calc software (developed by McGill University) recommended by the WHO was used to measure health disparities. The between group variance (BGV) of the absolute measure and the Theil index (T) of the relative measure were adopted for horizontal and vertical monitoring of China’s inequality in maternal and child health. The Slope function was used to analyse the BGV and T trends in various indicators between either the place of residence or gender from 2000 to 2013.

The absolute inequality reflected the degree of the difference in health between two subgroups and had the same dimensions as the health indicators. The relative inequality was the difference in the health proportion between the subgroups and was used to compare indicators with different dimensions. The variables were unordered for place of residence and gender; within the stratified indicators, both the BGV in the absolute inequality monitoring method and the T in the relative inequality monitoring method were used to assess the stratified variables based the equity of the unordered variables. Therefore, both methods were selected to measure and calculate the equity of maternal and child health outcome indicators in China. The T and BGV methods were used to consider and discuss equality in the studies of An R [25], Hajizadeh M [26], and An Q [27].

The BGV in the absolute inequality monitoring method is a commonly used statistic that measures the degree of discrepancy between groups and reflects the degree of deviation between a random variable and its mathematical expectation (i.e., average value). The variance is the average of the sum of the squares of the difference between individual data. In many practical problems, the study of variance (i.e., the degree of divergence) is a significant measure, with a larger BGV indicating greater inequality. The Theil index in the relative inequality monitoring method measures and calculates the relative inequality for the subgroups of the population that do not exist in the natural sequence. The T increases with an increase in the relative inequality; thus, a larger T indicates higher relative inequality without an upper limit. The Theil index shows the relative meaning rather than the absolute meaning. Therefore, T was mainly used to compare different indices in this study.

$$ \boldsymbol{B}\boldsymbol{G}\boldsymbol{V}={\displaystyle \sum_{\boldsymbol{j}-\boldsymbol{1}}^{\boldsymbol{J}}{\boldsymbol{P}}_{\boldsymbol{j}}}{\left({\boldsymbol{y}}_{\boldsymbol{j}}-\boldsymbol{\mu} \right)}^{\boldsymbol{2}} $$

P_j_ is the population size of group j, y_j_ is the average health level of group j, and μ is the average health level of the entire population.

$$ \boldsymbol{T}={\displaystyle \sum_{\boldsymbol{j}-\boldsymbol{1}}^{\boldsymbol{J}}{\boldsymbol{p}}_{\boldsymbol{j}}}{\boldsymbol{r}}_{\boldsymbol{j}}\boldsymbol{In}{\boldsymbol{r}}_{\boldsymbol{j}}\times \boldsymbol{1000} $$

P_j_ is the crowd weight of group j, and r_j_ is the indicator of the morbidity or health-related rate of group j.

## Results

### Developing trends in maternal and child health outcomes

**Fig. 1:**
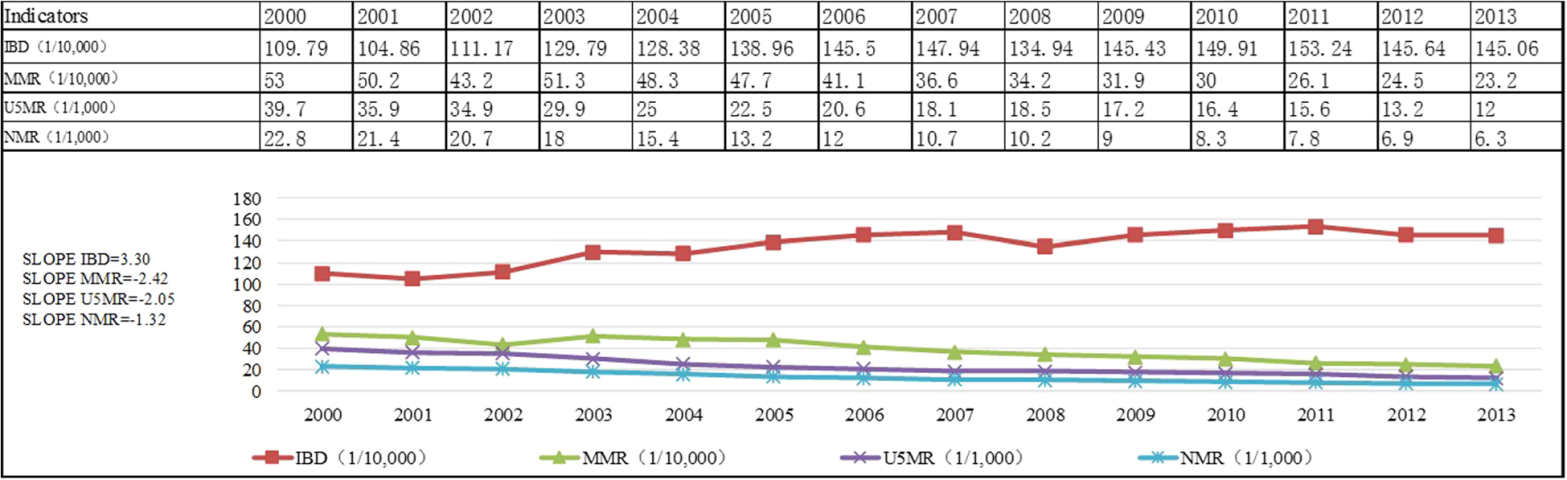
Trends in China’s maternal and child health outcomes from 2000 to 2013

Three indicators in the death outcome dimensions (MMR, NMR and U5MR) showed an annual declining trend. Conversely, the IBD showed an upward trend from 109.79 persons per 10,000 perinatal infants in 2000 to 145.06 persons in 2013; an IBD of 153.24 persons per 10,000 perinatal infants was found in 2011 (Fig. 1).

### Disparity trends in maternal and child health outcomes stratified by place of residence

**Fig. 2:**
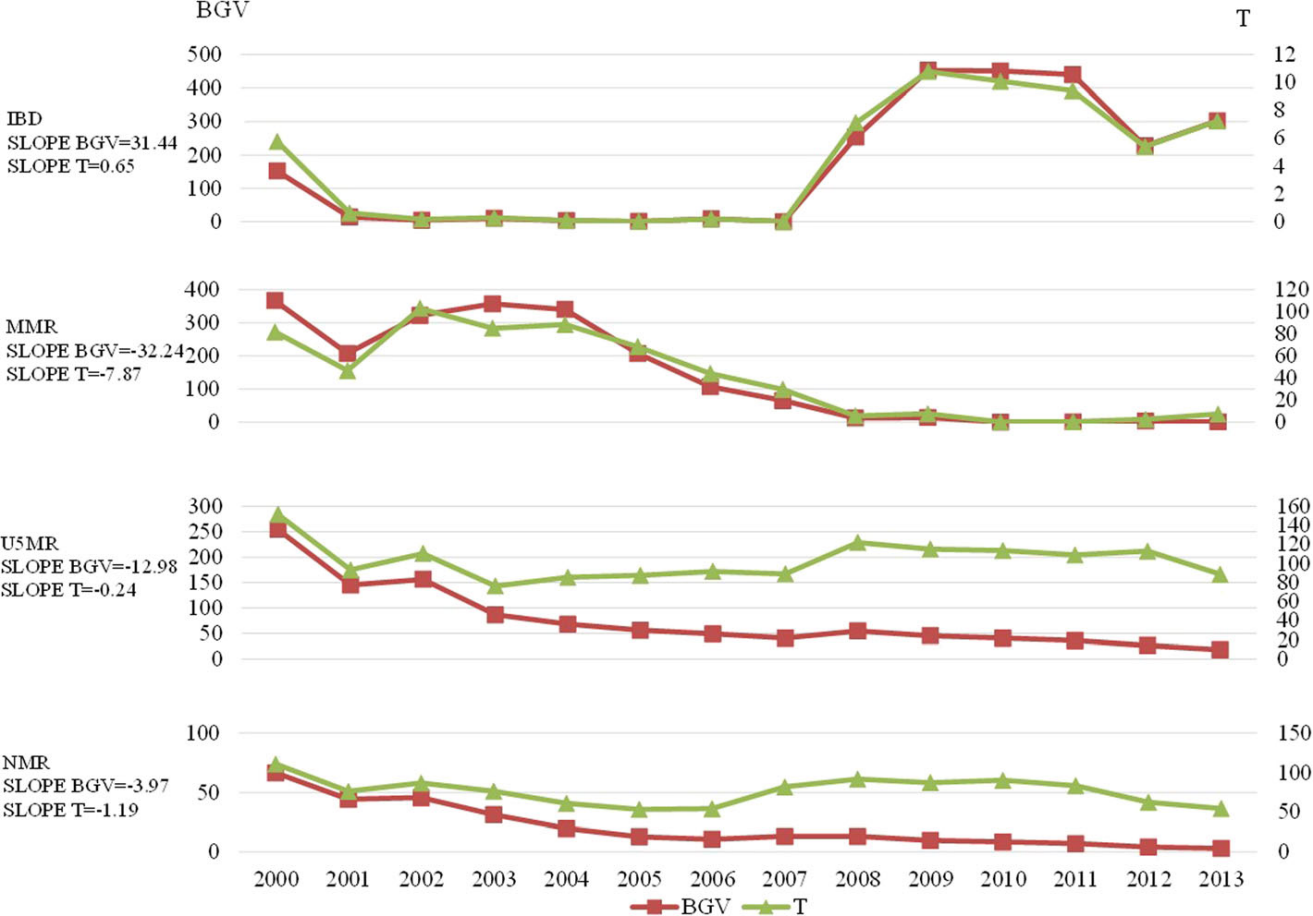
Disparity trends in China’s maternal and child health outcomes between urban and rural areas from 2000 to 2013

The following results were found in the comparison between urban and rural areas from 2000 to 2013: the BGV and T ranges for the IBD were [0.308, 452.413] and [0.007, 10.778], respectively, the BGV and T ranges for the MMR were [0.04, 366.723] and [0.022, 103.032], respectively, and the BGV and T ranges for the NMR were [3.24, 66.422] and [54.37, 110.764], respectively. The disparity of the MMR between the urban and rural areas was the lowest (T = 7.246) in 2013, whereas the disparity of the U5MR between urban and rural areas was the highest (T = 88.611). From 2000 to 2013, there was a decreasing trend in the disparity of all indicators with the exception of an upward trend in the disparity of the IBD between the urban and rural areas. The BGV (Slope BGV = -32.24) and T (Slope T = 7.87) of the MMR declined fastest and showed the largest range of decline, whereas the U5MR equality trend between the urban and rural areas showed slower improvement (Fig. 2).

### Disparity trends in maternal and child health outcomes stratified by gender

**Fig. 3:**
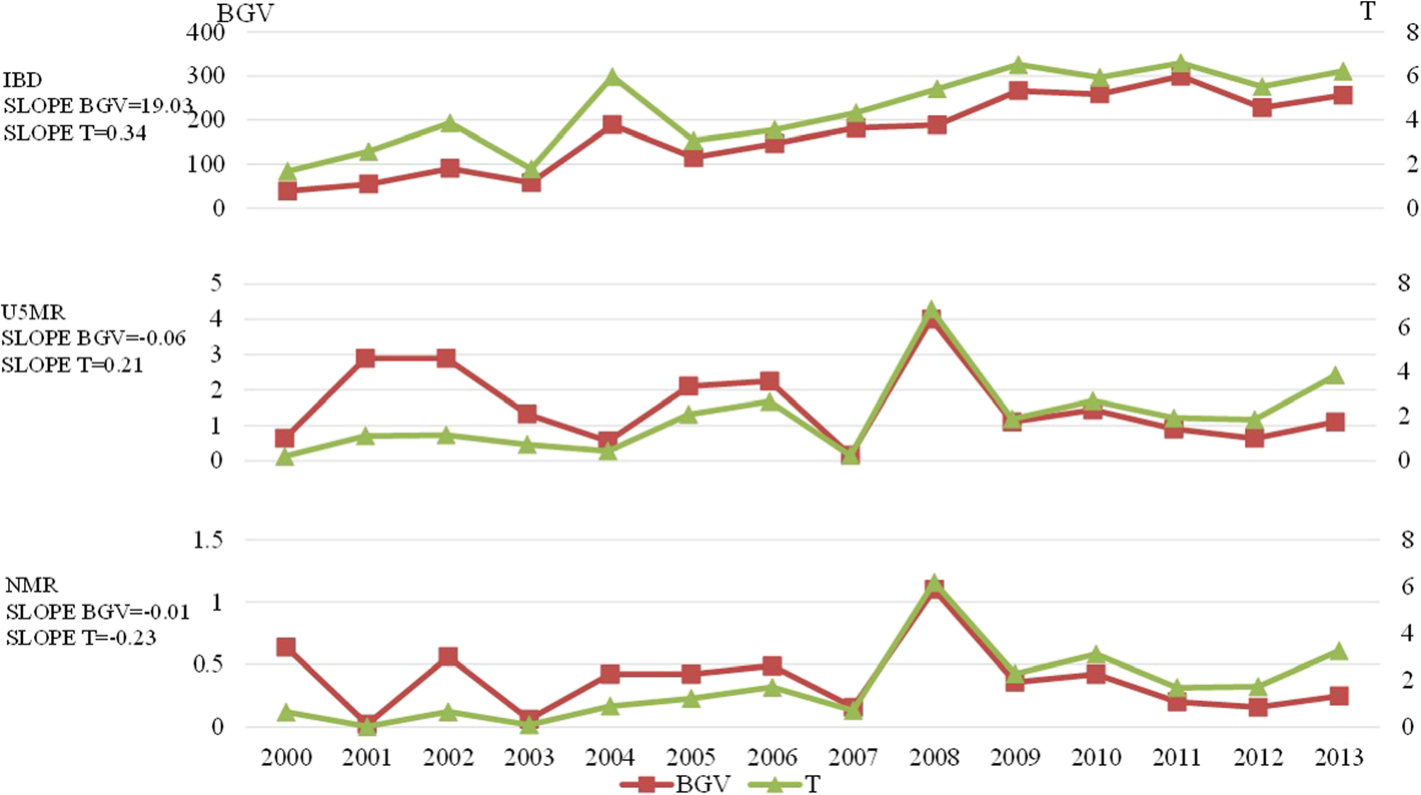
Gender disparity trends in China’s maternal and child health outcomes from 2000 to 2013

With the exception of the widening of the gender disparity in the IBD from 2000 to 2013, the development trends observed for gender disparity (Slope BGV = -0.06, Slope T = -0.21) in the U5MR and NMR were relatively stable (Slope BGV = -0.01, Slope T = 0.23). The BGV and T ranges for the IBD in the analysis stratified by gender from 2000 to 2013 were [38.875, 299.463] and [1.675, 6.595], respectively. The BGV and T ranges for the U5MR were [0.640, 4.000] and [0.210, 6.855], respectively. Finally, the BGV and T ranges for the NMR were [0.023, 1.102] and [0.025, 3.255], respectively. The gender disparity in IBD was largest in 2013 (T = 6.225), whereas the gender disparity in NMR was the lowest (T = 3.255) (Fig. 3).

### Disparity trends in maternal death causes based on place of residence

**Fig. 4:**
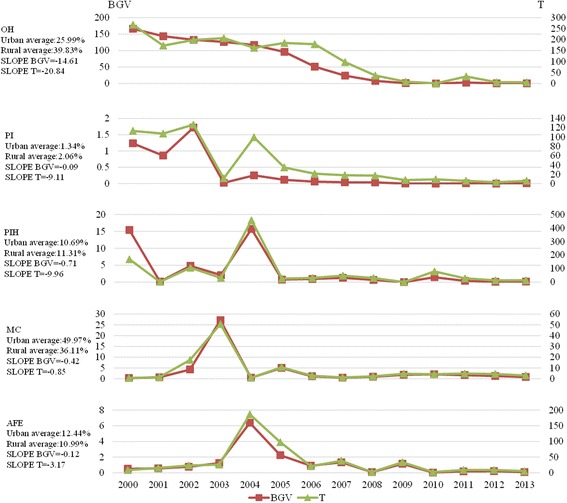
Disparity trends in maternal causes of death between urban and rural areas in China from 2000 to 2013

Based on the average values of various indicators, the sequence of urban maternal death among the five causes of death (OH, PI, PIH, MC and AFE) from 2000 to 2013 was as follows: MC (49.97%), OH (25.99%), AFE (12.44%), PIH (10.69%) and PI (1.34%). The sequence of rural maternal causes of death in 2000–2013 was as follows: OH (39.83%), MC (36.11%), PIH (11.31%), AFE (10.99%) and PI (2.06%) (Fig. 4).

Based on the overall developing trend from 2000 to 2013, the most obvious reduction in urban-rural differences in the maternal cause of death was OH (Slope BGV = -14.61, Slope T = -20.84). No significant changes were observed for the remaining indicators. With the exception of short-term fluctuations in individual years, the rural-urban differences in PI, PIH, MC and AFE were maintained at a low level. In 2013, the T of PIH was the largest (13.1), whereas the T of the other death causes was relatively small, with a distribution of [3.0, 13.1] (Fig. 4).

### Disparity trends in the causes of death in children under 5 years of age based on place of residence

**Fig. 5:**
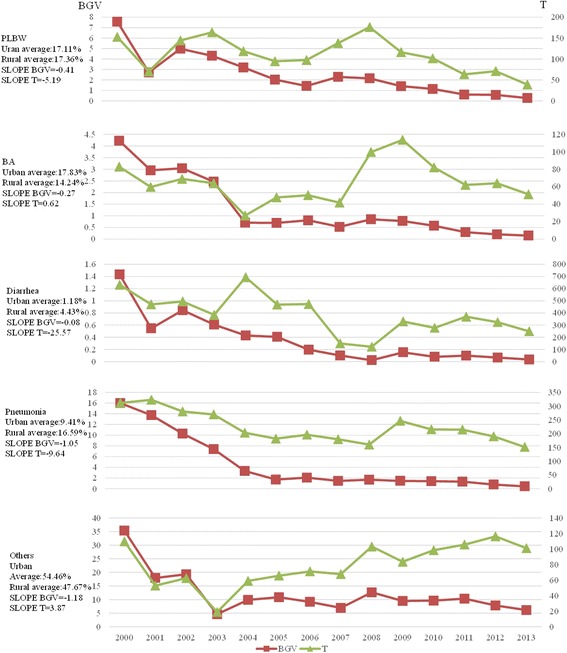
Disparity trends in causes of death of children under the age of 5 years between urban and rural areas in China from 2000 to 2013

From the average values, the sequence of the causes of death in children under 5 years of age in urban areas was as follows: other causes (54.46%), BA (17.83%), PBU (17.11%), pneumonia (9.41%) and diarrhoea (1.18%). The sequence of the causes of death in children under the age of 5 years in rural areas was as follows: other causes (47.67%), PBU (17.36%), pneumonia (16.59%), BA (14.24%) and diarrhoea (4.43%) (Fig. 5).

In 2013, a maximum rural-urban difference was found for diarrhoea in the different places of residence (T = 249.429). Conversely, the differences in PBU and BA were both relatively small (T = 38.945 and 51.244, respectively). The trend showed that the Slope BGV and T values for PBU, diarrhoea and pneumonia were less than 0, indicating that the urban-rural differences were narrow; however, the Slope BGV for the urban-rural differences in BA and the other causes of death trended in the opposite direction of the T values (Fig. 5).

### Disparity trends in the causes of death in children under 5 years of age based on gender

**Fig. 6:**
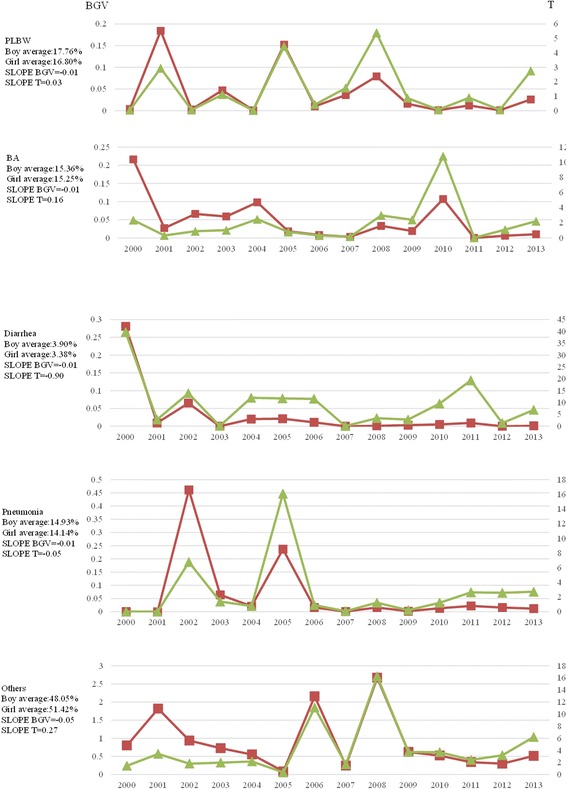
Gender disparity trends in causes of death of children under the age of 5 years in China from 2000 to 2013

Based on the average values, the sequence of the causes of death in boys under 5 years of age was as follows: other causes (48.05%), PBU (17.76%), BA (15.36%), pneumonia (14.93%) and diarrhoea (3.09%). The sequence of the causes of death in girls under 5 years of age was as follows: other causes (51.42%), PBU (16.80%), BA (15.25%), pneumonia (14.14%) and diarrhoea (3.38%). Thus, the same sequence was observed for boys and girls (Fig. 6).

In 2013, the gender-based T of PBU, BA, and pneumonia was approximately 2, whereas the gender-based T for diarrhoea and other causes of death was greater than 6. Based on the overall trend from 2000 to 2013, no obvious gender-based change across the five causes of death was found; the absolute values of the Slope T and BGV were both less than 1, although great fluctuation was observed in the values (Fig. 6).

## Discussion

### Comprehensive consideration of absolute and relative inequality monitoring methods

The results generally show agreement between the longitudinal equality trends in the various indicators represented by BGV and T, but some differences may exist. The BGV in PI indicated a decreasing longitudinal trend in urban-rural differences, whereas the T showed that the longitudinal trend in urban-rural differences increased. Additionally, the Slope BGV of the urban-rural differences in BA and other causes of death showed an opposing trend compared with that of the T value, which was similar to relevant research results. For example, Hajizadeh M and others used T and BGV to monitor equality in maternal and child services between six administrative regions and found that the T of skilled birth attendance in the different administrative regions decreased by 0.2 per year, whereas the BGV showed an upward trend [27].

### Overall improvement in the equality of maternal and child health outcomes in China

With the implementation and development of China’s major health projects, basic public health services and related maternal and child health special work throughout the country, China’s maternal and child health services are running at a high level [16]. Based on comparisons with historical data, the MMR, IBD, NMR and U5MR are decreasing annually. With the constant development towards equality in basic public health services [28], the equality of China’s maternal and child health outcomes has also improved. Regarding the equality between urban and rural areas from 2000 to 2013, improvement was found in the equity of three of the four outcome indicators. The degree of equality based on the place of residence and gender strata showed that the MMR increased overall and that the urban-rural differences in the U5MR and NMR decreased significantly. According to our interviews with officials in the health bureau to assess their actual health situations, various regions have rationally allocated maternal and child health resources and actively provided maternal and child health services in recent years. Furthermore, the efficiency has been enhanced and more attention has been paid to equity to achieve full coverage of basic services and wide coverage of special services. The most obvious change was the reduction in urban-rural differences.

The urban-rural differences in the construction mechanisms used to achieve equality in basic public health services in China’s maternal and child health field has narrowed. Specifically, the reduction in maternal and child health differences between urban and rural areas is mainly embodied in two aspects. First, the increased government investment in maternal and child health care projects in rural areas has effectively narrowed the difference in maternal and child health services between urban and rural residents. For example, our interview with health officials in Hubei Province showed that the “rural maternal subsidy for institutional delivery” from the central government to Hubei Province in 2014 represented an annual increase of 13%. Second, significant attendance by local government in the construction of maternal and child health care institutions in rural areas is intended to strengthen the coordination of urban and rural maternal and child health care. For example, apart from the increasing financial subsidies and material support for maternity and child care institutions in rural areas, the local government in Datong County, Qinghai Province, has paid more attention to the selection of personnel for the maternity and child care institutions in these areas. Datong County has gradually established three levels of professional training and support mechanisms for maternal and child health hospitals at the county (city), township, and village clinic (institution) levels. Every year, superior maternal and child health care institutions will send expert groups to provide operational guidance and related training to inferior maternal and child health care institutions. Regarding the increase in support for rural areas, especially the institutions at and below the county level, the government should increase support to areas where the socioeconomic status is relatively low (especially remote and lacking rural areas). This approach may improve or increase related maternal and child health (MCH) services, initiate projects to develop maternal and child health care institutions in these areas, transfer project-promoting financial support to take advantage of multiple forms, such as transfer payments, develop maternal and child support projects to explore the long-term development mechanism of maternal and child health institutions and consolidate the achievement of the equalization of basic public health services.

### Reasons for the deterioration of the equality in IBD

Of the evaluated indicators, IBD showed the highest overall level and a deeper degree of inequality. The increasing trend in IBD and the enlarged difference between groups may be due to technological progress, such as the development of medical examination technology, which facilitates the detection of birth defects. Furthermore, the development of statistical methodology has allowed more recording of birth defect statistics.

In terms of inequality, the difference between urban and rural areas increased by 40% between 2000 and 2013. From the results, we noted that the inequality in IBD reached its peak after a compulsory premarital check was cancelled in 2003. Therefore, the decline in premarital medical examinations may also be a cause of the increased inequality in IBD. A premarital medical check-up plays an important role and is the primary threshold for the prevention and control of birth defects in China [29]; specifically, there is a greater preventive impact of premarital medical check-ups in rural, remote, and socio-economically underdeveloped areas [30]. Additionally, the recognition and support of local governments for premarital medical examinations are more significant for the reduction in IBD and enhancement of IBD equality [31]. Attention should be paid to the function of primary preventive methods to control birth defects, including premarital examinations, while continuously implementing functions such as the “pre-pregnancy eugenic health check” and “neonatal screening in poor areas”. Depending on the condition, regions can also expand the services of these projects based on their own needs and provide partially paid services to maximize their effectiveness.

### Search for breakthroughs in urban-rural differences in mortality based on the analysis of the equality of the causes of death

Cause of death statistics are important epidemiological data and are the main source of information for the formulation of health policy and the determination of health resource allocation and disease intervention focuses [32]. Approximately 50 years of historical data are available in the death cause registration report in China [33]. At present, the four main case management systems for the national death cause registration report are as follows: Death Cause Registration System of the Ministry of Health [34], National Disease Surveillance Points System, the National Maternal and Child Health Monitoring System (NMCHMS) [35] and the National Death Cause Registering and Reporting System (i.e., the system described herein). Most of the documentation on maternal mortality and deaths in children under 5 years of age is sourced from the National Maternal and Child Health Monitoring System (as in this study).

The different sequences in the causes of death reflect an unfair outcome. The results of this study show similar sequences in the causes of death within the U5MR for different genders, whereas the U5MR and MMR differ between urban and rural areas. When comparing Figs. 2 to 3, we found that the gender differences in the IBD, U5MR, and NMR for the same year were smaller than the urban and rural differences; additionally, no gender difference is found in pregnant women. Therefore, this study focused on the analysis of urban-rural differences in an effort to reduce the overall differences; this approach was likely to reduce the urban-rural differences in maternal and child health.

To reduce the number of deaths in children under 5 years of age in medical institutions, attention should be paid to perinatal diseases, and effective measures, such as neonatal asphyxia resuscitation technology, should be promoted. Respiratory diseases should also be considered, especially pneumonia, which is not only a common disease in children under the age of 5 years but is also a common cause of death in children in medical institutions [36]. Therefore, regulating the use of antibiotics is essential. Additionally, more attention should be placed on cardiac congenital malformations, the physical examination process should be standardized for children, and dedicated auscultation should be performed to find cardiac congenital malformations as early as possible. Both the timely treatment of children and the selection of an appropriate time for cardiac surgery are effective measures.

From the survey of global maternal death causes, OH is the major cause of maternal mortality in Asia [37]. The OH equality data from 2000 to 2013 illustrate significant improvement in the urban-rural gap (from 267.7 to 5.5). In this study, the major causes of OH were uterine inertia, placental factors, damage to the uterine and birth canal, and coagulation defects [38]. The recent awareness, prevention and control of OH through constant and strengthened rural basic medical and health institutions in China and symptomatic treatment training of the causes of OH have enabled rural maternal health care institutions to implement maternal referrals in a timely manner and administer effective treatment [39]. These improvements have gradually narrowed the urban-rural gap. Additionally, the urban-rural gap in deaths caused by PIH is related to the socioeconomic status, extent of education, and living conditions of pregnant women with and without PIH, among others. Therefore, China’s health sectors actively provide grass-roots maternal health services to promote equality in public health services, increase publicity efforts, encourage rural maternal women to receive timely antenatal examinations and increase the number of antenatal examinations; overall, this work will effectively control PIH and other common diseases during pregnancy.

### Limitations

The data in this study were mainly obtained from the official authority data of China’s Maternal and Child Health Surveillance Network, and accepted sensitive outcome indicators were selected. However, the network’s lack of individual cases and structural and procedural indicators may lead to a deficiency in the comprehensiveness of the data. Although this lack could be a limitation of this study, the purpose of this study was to provide the government with a policy basis through a macro-level equality analysis; therefore, the use of official data had an appropriate effect. In the future, we will perform a case study based on a macro-level equality analysis and consider building a more comprehensive index system to enrich relevant research results.

## Conclusions

From 2000 to 2013, general improvement in the equality of maternal and child health was found in China. Within the overall performance, greater differences were found between urban and rural areas than between genders. However, there is still a need for improvement. In particular, there is an increasing gap in IBD between urban and rural areas. Therefore, seizing crucial links, actively exploring appropriate technology to reduce the overall IBD and improving the range of inequality issues are essential. Additionally, improved health equality must be consolidated and the urban-rural gap in MMR and U5MR must be narrowed by focusing on differences in the causes of death between urban and rural areas.

## Abbreviations

AFE: Amniotic fluid embolism
BA: Birth asphyxia
BGV: Between group variance
IBD: Incidence of birth defects
MC: Medical complications
MDGs: Millennium development goals
MMR: Maternal mortality rate
NMCHMS: National maternal and child health monitoring system
NMR: Neonatal mortality rate
OH: Obstetric hemorrhage
PBU: Premature birth with underweight
PI: Puerperal infection
PIH: Pregnancy induced hypertension
SDGs: Sustainable development goals
T: Theil index
U5MR: Under 5 mortality rate
WHO: World Health Organization
